# Novel Integration of Extracorporeal Membrane Oxygenation and Continuous Renal Replacement Therapy in Pediatric Patients With Severe Burns: A Case Report

**DOI:** 10.7759/cureus.102924

**Published:** 2026-02-03

**Authors:** Hiroshi Matsuoka, Tomohiro Abe, Tetsu Yonaha, Takeshi Yano, Masahiko Taniguchi, Takehiko Nagano, Isao Tsuneyoshi, Hidenobu Ochiai

**Affiliations:** 1 Department of Emergency and Critical Care Medicine, University of Miyazaki Hospital, Miyazaki, JPN; 2 Department of Emergency and Critical Care Medicine, University of Miyazaki Hospital, Miyazaki city, JPN; 3 Cardiovascular Biology Research Program, Oklahoma Medical Research Foundation, Oklahoma City, USA; 4 Department of Anesthesiology and Intensive Care, University of Miyazaki Hospital, Miyazaki, JPN

**Keywords:** acute kidney injury, burns, extracorporeal membrane oxygenation, renal replacement therapy, respiratory distress syndrome

## Abstract

Acute respiratory distress syndrome (ARDS) and acute kidney injury (AKI) represent critical complications that significantly burden the management of pediatric patients with extensive burns. We present a case of a four-year-old boy who sustained severe burns covering 56% of his total body (Burn Index 29). The patient developed ARDS on Day 3, which led to the initiation of venovenous extracorporeal membrane oxygenation (ECMO) on Day 5. Following the subsequent development of AKI and fluid overload, continuous renal replacement therapy (CRRT) was introduced on Day 6. To overcome the limitations of vascular access common in pediatric patients, a novel integrated approach was employed by connecting the CRRT circuit to a unique bypass between the ECMO limbs. This integrated system allowed for seamless treatment and effective fluid management, resulting in the resolution of pulmonary edema and a significant improvement in respiratory function. The patient was successfully weaned from ECMO on Day 10 and CRRT on Day 13, eventually being discharged on Day 90 following multiple skin grafting procedures. This case highlights that the integration of CRRT into an ECMO bypass line is a simple, safe, and effective modality for the life-saving treatment of pediatric severe burns complicated by multi-organ failure.

## Introduction

Severe burn injuries in children are a clinical challenge, particularly in the toddler and preschool age groups, as they are associated with a relatively high mortality rate [1]. Severe burns cause profound inflammation that leads to critical organ dysfunction, including respiratory, circulatory, and renal failure. Unlike burns in adult patients, pediatric burns can have unique characteristics and complications, such as smaller body size, limited vascular access, and narrow optimal therapeutic windows [2], although the mortality rate of pediatric patients with severe burns is lower than that of adults [3].

To support these failing organs, specialized life-support systems are required. Extracorporeal membrane oxygenation (ECMO) functions essentially as an artificial lung or heart-lung machine; in its venovenous modality, it provides oxygenation support, whereas the venoarterial modality also supports systemic blood flow to assist a failing heart. ECMO is a potential treatment option for adult patients with severe burns, offering a lifeline for those experiencing critical respiratory and circulatory failure [4]. Similarly, renal replacement therapy (RRT) acts as an artificial kidney, managing not only fluid, electrolyte, and metabolic balance but also helping to reduce systemic inflammation [5]. However, combined ECMO and RRT in burn patients with respiratory, circulatory, and renal dysfunction is challenging due to concerns regarding limited vascular access and circuit configuration. This challenge is particularly underscored in pediatric cases, where a significant knowledge gap remains concerning the optimal method of integrating these systems to address the unique anatomical constraints of children. In this paper, we present the case of a child with severe burns who developed respiratory dysfunction and was successfully treated with a combination of ECMO and RRT. The RRT circuit was connected to a unique bypass between the ECMO limbs, which allowed for well-tolerated, event-free treatment and ultimately saved the patient’s life.

## Case presentation

A four-year-old boy with severe burns was transported to our hospital after falling into a cauldron approximately 70 cm deep containing oil heated to nearly 100 °C. His father immediately pulled him out and cooled the burns with water. He had no significant medical history and was not taking any medications at home.

Upon arrival, his vital signs were stable, with a body temperature of 35.1 °C, blood pressure of 113/56 mmHg, and heart rate of 113 bpm. Physical examination revealed erosions on the face, trunk, upper limbs, and both thighs, with near-complete epidermal peeling and redness. The patient was diagnosed with severe burns covering 56% of the total body surface area, with a Burn Index of 29. The patient was admitted to our intensive care unit for management, including initial fluid resuscitation, mechanical ventilation, and wound care on an air-fluidized bed. Initial fluid resuscitation was performed according to Advanced Burn Life Support guidelines, successfully achieving a urine output exceeding 2 mL/kg/hour. Although the patient had only mild laryngeal edema and no evidence of airway burns, he was preemptively intubated due to concerns about fluid overload and impending respiratory deterioration.

On the morning of Day 3, the patient's oxygen saturation (SpO_2_) dropped to 60%-70%. A chest X-ray revealed atelectasis of the right upper lobe. Given the weight increase from a baseline of 14.6 to 18.6 kg on Day 3, pulmonary edema was considered an additional contributor to respiratory dysfunction. Although his SpO_2_ improved after adjusting the ventilator to a moderate positive end-expiratory pressure (PEEP) of 13 cm H_2_O, the patient developed bradycardia and hypotension in the afternoon, requiring cardiopulmonary resuscitation, which successfully restored spontaneous circulation. On Day 4, the patient's respiratory status significantly deteriorated. Despite ventilator settings of FiO_2_ 1.0 and PEEP at 20 cmH_2_O, oxygenation was severely compromised with a PaO_2_/FiO_2_ ratio of 69, leading to a diagnosis of acute respiratory distress syndrome (ARDS).

**Figure 1:**
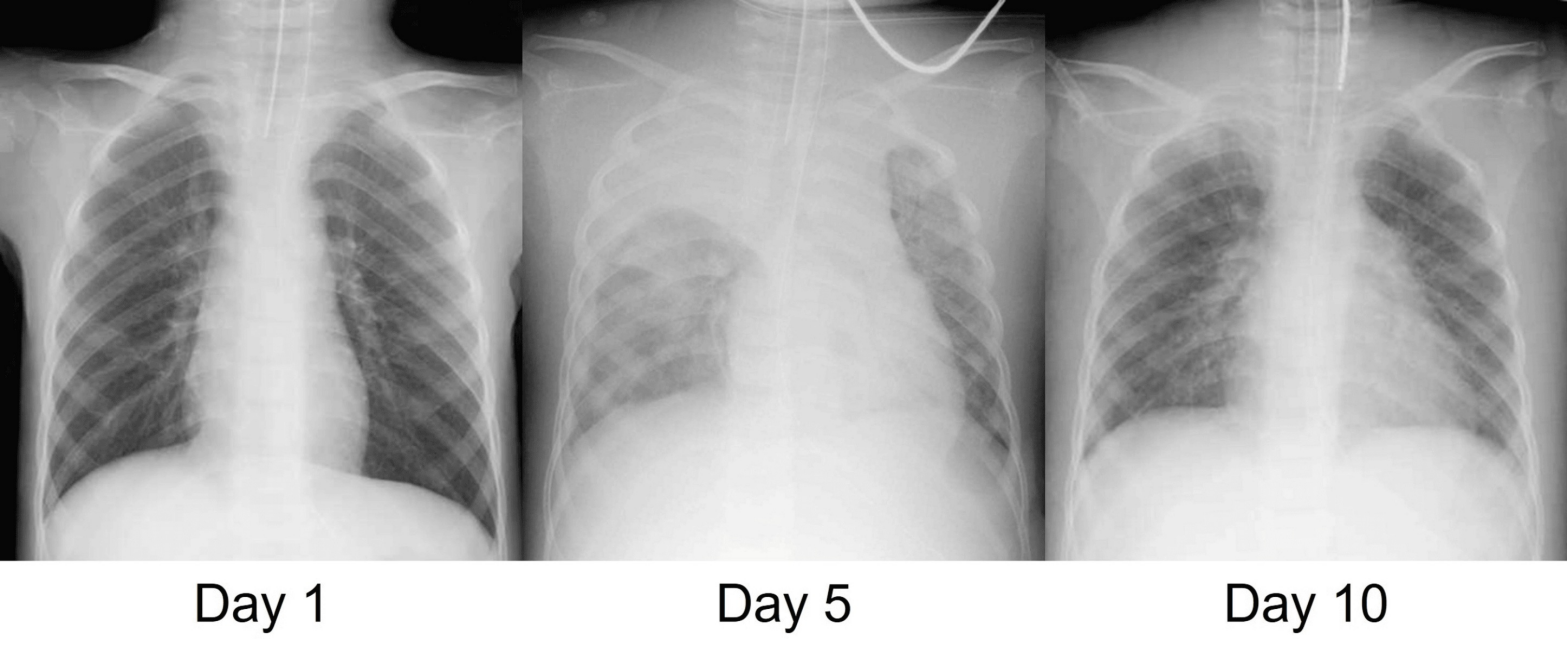
Chest X-ray on Days 1, 5, and 10. Day 5 highlights severe pulmonary edema and atelectasis, which compromised respiratory function and necessitated the initiation of ECMO. Day 10 demonstrates significant resolution of these findings following the initiation of integrated CRRT, which allowed for precise fluid removal, resulting in the improvement of the patient’s respiratory status. ECMO, extracorporeal membrane oxygenation; CRRT, continuous renal replacement therapy

On Day 5, a chest X-ray revealed pulmonary edema and atelectasis in the right upper lobe (Figure 1).

**Figure 2:**
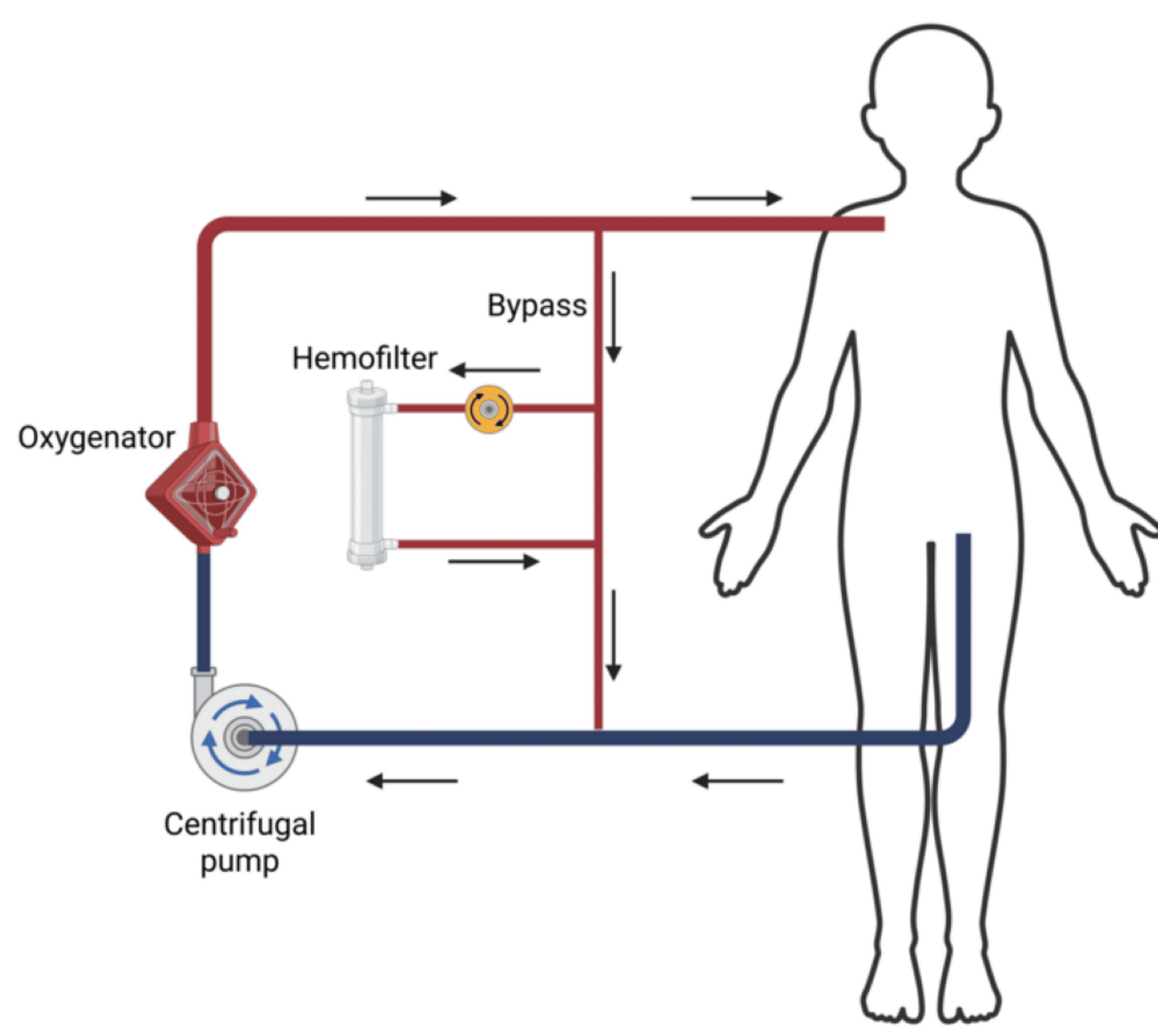
Schematic diagram of circuit configuration integrating ECMO and CRRT. A direct bypass was established between the return and drainage cannulas, enabling partial recirculation of reinfused blood through the membrane oxygenator to enhance oxygenation. The CRRT circuit was connected to the ECMO bypass between the return and drainage cannulas. Image created by the authors using BioRender.com. ECMO, extracorporeal membrane oxygenation; CRRT, continuous renal replacement therapy

Due to severely compromised respiratory function, venovenous ECMO was initiated (centrifugal pump: MixFlow 3, JMS Co. Ltd., Hiroshima, Japan; oxygenator: BioCube 2000, NIPRO Corp., Osaka, Japan), with drainage from the left femoral vein and return to the right subclavian vein using 14 Fr cannulas (Fem-Flex II Femoral Venous and Arterial Cannulas, Edwards Lifesciences Corp., Irvine, CA). An ECMO flow of 2.5 L/minute (pump speed: approximately 2,500 rpm) was maintained by adjusting cannula positioning and optimizing volume status via intravenous fluid administration and vasopressor support (noradrenaline at approximately 0.05 µg/kg/minute). To enhance oxygenation, a direct bypass was created from the return to the drainage cannula, allowing a portion of the reinfused blood to recirculate through the membrane oxygenator for additional oxygenation. These attempts achieved stable hemodynamics, with blood pressure maintained within the range of 100-120/40-60 mmHg. On Day 6, despite maintaining a urine output of approximately 600 mL/day (~1.7 mL/kg/h), the patient developed progressive fluid overload (exceeding 30% weight gain from baseline) and worsening pulmonary edema that compromised respiratory status. Although the patient did not strictly meet the KDIGO criteria for acute kidney injury (AKI) based on serum creatinine or urine output at that time, continuous renal replacement therapy (CRRT) using continuous hemodiafiltration (CHDF) was initiated (system: ACH-Σ, Asahi Kasei Medical Co., Ltd., Tokyo, Japan; hemofilter: UT-700S, NIPRO Corp., Osaka, Japan). Given the difficulty of securing dedicated vascular access due to severe burns, the CHDF circuit was integrated into the ECMO bypass between the drainage and return cannulas. Specifically, blood was accessed from the post-pump side and returned to the pre-pump side of the ECMO circuit (Figure 2).

Throughout the treatment, the access pressure was maintained at 65-90 mmHg, and the return pressure was carefully monitored and kept between 20 and 35 mmHg to prevent excessive suction from the ECMO pump. Regarding anticoagulation, systemic heparinization was maintained for the ECMO with a target activated partial thromboplastin time (APTT) of 1.5 times the upper limit of normal, while nafamostat mesylate was additionally administered into the CHDF circuit. This regional anticoagulation strategy, combined with the optimized pressure management, maintained a stable transmembrane pressure of 40-55 mmHg, facilitating consistent circuit performance and minimizing interruptions caused by clotting or pressure alarms.

Following CHDF initiation, the patient's fluid balance improved through ultrafiltration, leading to resolution of pulmonary edema (Figure 1) and subsequent improvement in respiratory function (Figure 2).

**Figure 3:**
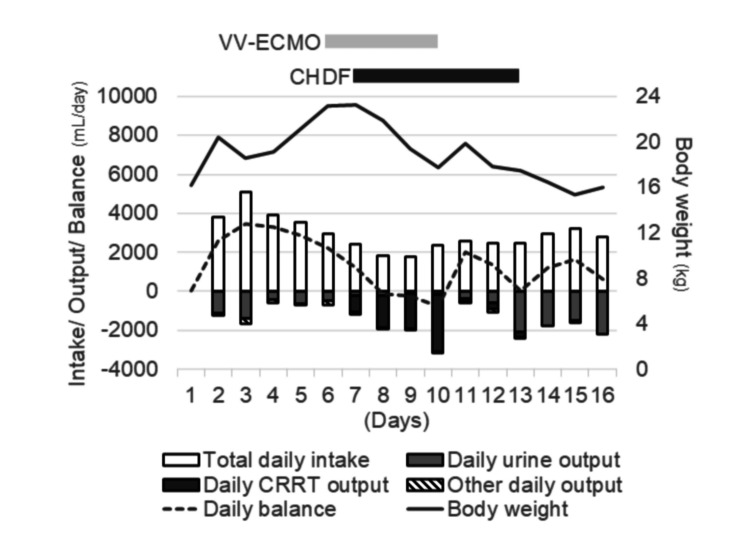
Daily fluid balance during the clinical course in this case. Urine output (blank columns) remained insufficient until Day 5, resulting in a persistently positive daily fluid balance (dotted line) and increased body weight (black line), which contributed to respiratory deterioration and prompted the initiation of VV-ECMO. On Day 6, CHDF was initiated, and fluid removal (dark columns) reduced the daily fluid balance, which became negative after Day 8. Body weight subsequently decreased, allowing for ECMO withdrawal on Day 10. CHDF was continued until Day 13, when urine output stabilized at approximately 2 L/day, after which it was discontinued. Body weight remained stable at approximately the same level as on Day 1. ECMO, extracorporeal membrane oxygenation; CRRT, continuous renal replacement therapy; CHDF, continuous hemodiafiltration

ECMO was successfully discontinued on Day 10. CHDF was continued until adequate urine output was restored and fluid balance stabilized, after which it was discontinued on Day 13 (Figure 3).

Following systemic stabilization, the first surgery was performed on Day 16, involving debridement of the back, buttocks, right upper limb, left upper arm, and anterior chest. Skin grafting was performed using skin harvested from the scalp and left lower leg, with mesh grafts applied to the back, patch grafts to the buttocks, and sheet grafts to the dorsum of the hand. An artificial dermis was applied to the anterior chest. Mechanical ventilation was discontinued on Day 24. A second surgery was performed on Day 28, consisting of debridement of the anterior chest and upper limbs, followed by cultured epidermis grafting. On Day 39, the patient was transferred from the ICU to the general ward, with less than 10% of the body surface area affected by burn ulcers. The patient underwent an additional surgery on Day 72. Following continued postoperative care, nearly all ulcerated areas healed, and the patient was discharged on Day 90.

## Discussion

This report describes the case of a pediatric patient with severe burns who developed respiratory dysfunction and was successfully treated with venovenous ECMO. Although the patient also developed acute kidney injury and fluid overload during ECMO, combined therapy with CRRT restored homeostasis. To the best of our knowledge, this is the first pediatric case of its kind reported in the literature.

Respiratory ECMO is a well-established treatment option for patients with severe respiratory dysfunction. Among pediatric patients, respiratory ECMO is associated with the lowest mortality in those with severe burns, compared to those with other underlying conditions. Thus, respiratory ECMO is considered a treatment option for patients with respiratory failure unresponsive to conventional therapies. Approximately 70% of patients receiving ECMO develop acute kidney injury and fluid overload, both associated with high mortality and representing the most common indications for CRRT [6,7]. In pediatric patients on ECMO, fluid overload is linked to increased mortality, and early initiation of CRRT may improve outcomes by controlling fluid balance [6].

The connection modality remains a major challenge in combined CRRT and ECMO therapy. There are two major approaches for establishing CRRT in patients undergoing ECMO. The parallel approach uses an independent CRRT circuit separate from the ECMO circuit, whereas the integrated approach incorporates the CRRT circuit directly into the ECMO system [8,9]. The integrated approach has the advantage of requiring less vascular access, thereby reducing the risk of catheter-related complications. This is particularly important in patients with severe burns, given the limited area of intact skin available for catheter placement. The primary concern regarding the integrated approach is how the two circuits are connected. Current integrated methods typically involve connecting the CRRT circuit either pre-pump or post-pump within the ECMO system. Connecting CRRT pre-pump carries a risk of air entrapment and potential air embolism if significant air leaks occur. Conversely, post-pump connection, the most common integrated system, exposes the CRRT system to positive pressure, which can lead to treatment failure due to intra-circuit pressure imbalances and hemofilter damage [9]. Inline hemofilters are a simple alternative; however, they lack precise volumetric and pressure control. Our unique bypass serves as a pressure-buffering system that mitigates excess post-pump pressure while allowing precise control of ultrafiltration. This configuration overcomes the technical limitations of standard integration methods, ensuring more reliable and event-free CRRT for children with limited vascular access.

Beyond burn care, our bypass-integrated approach offers versatility across the pediatric spectrum. It is particularly advantageous for neonates with extremely limited vascular access and for larger children requiring stable circuit pressures. This method is highly applicable to various conditions necessitating multi-organ support, such as severe sepsis, cardiogenic shock, and congenital heart disease, where fluid management is critical. By preserving limited access sites while ensuring circuit stability, this configuration provides a robust solution for integrated extracorporeal therapies in various critically ill pediatric patients.

Although our method of establishing the CRRT circuit via ECMO bypass did not result in complications or treatment failure, several potential concerns warrant consideration. First, rapid fluctuations in intra-circuit pressure may lead to treatment failure; therefore, continuous and careful pressure monitoring is essential. Second, there is a potential risk of intra-circuit thrombosis, particularly within the bypass line, which must be closely monitored when using this method.

## Conclusions

Pediatric patients with extensive burns face a high mortality risk when complicated by ARDS and AKI. This case demonstrates that combined ECMO and CRRT is a vital rescue modality when conventional management fails. Our integrated approach - connecting the CRRT circuit via a unique bypass between the ECMO limbs - effectively overcomes the challenge of limited vascular access in small children. This configuration provides a stable platform for continuous therapy without significant interruptions from circuit clotting or pressure-related alarms. Although careful monitoring for intra-circuit pressure and thrombosis is essential, this simple and safe configuration offers a life-saving intervention for critically ill children with multi-organ failure, extending its utility beyond burn-specific cases. However, further multicenter studies are warranted to validate the safety of this configuration and to standardize it for broader adoption across a wider range of pediatric patients, including larger children.
